# Collection of non-meconium stool on fecal occult blood cards is an effective method for fecal microbiota studies in infants

**DOI:** 10.1186/s40168-017-0333-z

**Published:** 2017-09-05

**Authors:** Wendy S.W. Wong, Nicole Clemency, Elisabeth Klein, Marina Provenzano, Ramaswamy Iyer, John E. Niederhuber, Suchitra K. Hourigan

**Affiliations:** 1 Inova Translational Medicine Institute (ITMI), 3300 Gallows Road, Claude Moore Bldg, 2nd Floor, Falls Church, VA 22042 USA; 2 Inova Children’s Hospital, 3300 Gallows Road, Falls Church, VA 22042 USA; 3 Pediatric Specialists of Virginia, 3023 Hamaker Court, Suite 600, Fairfax, VA 22031 USA; 4 Johns Hopkins School of Medicine, 733 N Broadway, Baltimore, MD 21205 USA

**Keywords:** Stool, Meconium, Microbiota, Fecal occult blood card, Children, Storage

## Abstract

**Background:**

Effective methods are needed to collect fecal samples from children for large-scale microbiota studies. Stool collected on fecal occult blood test (FOBT) cards that can be mailed provides an effective solution; however, the quality of sequencing resulting from this method is unknown.

The aim of this study is to compare microbiota metrics of 16S ribosomal RNA (rRNA) gene sequencing from stool and meconium collected on FOBT cards with stool collected in an Eppendorf tube (ET) under different conditions.

**Methods:**

Eight stool samples from children in diapers aged 0 month–2 years and three meconium samples were collected and stored as follows: (1) ≤ 2 days at room temperature (RT) in an ET, (2) 7 days at − 80 °C in an ET, (3) 3–5 days at RT on a FOBT card, (4) 7 days at RT on a FOBT card, and (5) 7 days at − 80 °C on a FOBT card. Samples stored at − 80 °C were frozen immediately. Each specimen/condition underwent 16S rRNA gene sequencing with replicates on the Illumina MiSeq. Alpha and beta diversity measures and relative abundance of major phyla were compared between storage conditions and container (ET vs. FOBT card), with pairwise comparison between different storage conditions and the “standard” of 7 days at − 80 °C in an ET and fresh stool in an ET.

**Results:**

Stool samples clustered mainly by individual diaper (*P* < 10^−5^, Adonis), rather than by storage condition (*P* = 0.42) or container (*P* = 0.16). However, meconium samples clustered more by container (*P* = 0.002) than by individual diaper (*P* = 0.009) and storage condition (*P* = 0.02).

Additionally, there were no differences in alpha diversity measures and relative abundance of major phyla after Bonferroni correction between stool stored on a FOBT card at RT for 7 days with stool stored in an ET tube at − 80 °C; differences in alpha diversity were seen however when compared to fresh stool in an ET. Overall, based on the intraclass correlation coefficient (ICC), the different storage containers/conditions are reliable in preserving the microbial memberships and slightly less reliable in preserving the alpha diversity and relative microbial composition of infant stool.

**Conclusions:**

Acknowledging certain limitations, FOBT cards may be a useful tool in large-scale stool microbiota studies in children requiring outpatient follow-up where only small amounts of stool can be obtained, but should not be used when studying meconium.

**Electronic supplementary material:**

The online version of this article (10.1186/s40168-017-0333-z) contains supplementary material, which is available to authorized users.

## Background

The role of the intestinal microbiota in human health and disease is becoming increasingly recognized, with a rapidly growing number of studies in this area. To date, many studies have been small scale and cross-sectional; to fully understand the role of the intestinal microbiota in disease states and health, large-scale prospective longitudinal studies are required. For these large-scale studies to be successful, a cost-effective and convenient way for fecal sampling of participants is needed that can produce microbiota sequencing results comparable to conventional methods. Moreover, for children in diapers, a method requiring only small amounts of fecal material is optimal due to the issues of small stool volumes and difficulties in obtaining adequate stool from the diaper.

Collection of stool by fecal occult blood test (FOBT) cards represents a method that fulfills the criteria for the following reasons: (1) With the often very small amounts of fecal material found in diapers of young children and with the feces sometimes being mostly soaked into the diaper, it is possible to scrape enough stool to apply to an FOBT card but not enough to put in an Eppendorf tube (ET); this is regularly found even in the lab setting with stool received on diapers. (2) The cards are commercially available and inexpensive. (3) The flat shape and small size makes the card easier to include in packages to be sent to participants compared with bulkier containers such as ETs. (4) Because the cards are widely used in the pediatric gastroenterology clinic and for colorectal cancer screening, potential participants and parents might be more accepting of collecting samples in this way. (5) FOBT cards have an accompanying small FDA-approved mailing envelope which can be sent by regular mail, which is much more convenient for the families to return and inexpensive compared to the US Postal Service regulations for stool samples in containers such as ETs (triple packaged and within a secondary container). However, there have been limited studies examining the microbiota sequencing results in terms of stability in different conditions, reproducibility, and ability to preserve the microbiota signature of stool collected on FOBT cards compared to more traditional methods of sequencing from fresh or frozen stool. Small studies in healthy adults have demonstrated that overall, collection of stool by FOBT card is a reasonable collection method for 16S ribosomal RNA (rRNA) gene sequencing [1, 2]; however, other adult studies have shown that storage conditions can significantly influence microbiota profiles by 16S rRNA gene sequencing [3].

There are few studies evaluating the use of FOBT cards as a collection method for fecal microbiota studies in children. This method may produce different results from adults, and especially in young children, for many reasons including the rapidly developing dynamic microbiota in the first few years of life with a generally lower microbiota diversity and differing composition compared with adults [4], the often small amount of stool available, and the need to scrape stool from a diaper onto the card. Moreover, there has been an increasing number of studies looking at the microbiota composition of meconium [5–7], which greatly differs in microbiota composition [8] and ease of DNA extraction from stool; the effect of storage of meconium from FOBT cards for sequencing is unknown.

Therefore, in this study, we aimed to compare 16S rRNA gene sequencing results from stool and meconium stored on a FOBT card versus in an ET under different conditions. Specifically, we aimed to test whether meconium and stool samples stored with FOBT cards at room temperature for up to 7 days (to replicate mailing conditions) would be a viable alternative to those samples stored in ETs frozen immediately at − 80°C (the current standard for many studies).

## Methods

### Sample collection and storage conditions

Infants and children were enrolled in an IRB-approved longitudinal genomic study at the Inova Translational Medicine Institute. A diaper containing stool or meconium (the first stool passed) was collected from participants. The stool from the diaper was then divided into the following storage conditions before DNA extraction: (1) ≤ 2 days at room temperature (RT) in an ET, (2) 7 days at − 80 °C in an ET, (3) 3–5 days at RT on a FOBT card, (4) 7 days at RT on a FOBT card, and (5) 7 days at − 80 °C on a FOBT card. Hemoccult II SENSA cards® (Beckman Coulter, CA) were used. Up to 0.1 g of stool or meconium was applied to each square of the FOBT card which contains two squares (range 0.013 g–0.1 g per square). The differing lengths of storage on a FOBT card held at room temperature were to replicate possible different conditions if participants were to mail samples from home. The storage at − 80 °C in an Eppendorf tube was to represent the standard used in most studies to date [3, 5, 6]. The storage for 7 days at − 80 °C on a FOBT card was to examine whether FOBT cards could be frozen before DNA extraction as it is often more convenient for labs to extract the DNA in batches rather than separately for each sample received. The samples stored at − 80 °C were frozen immediately after they were aliquoted. To assess for reproducibility, if enough stool or meconium was available from a single diaper, multiple aliquots for each storage condition were stored.

### Sample extraction

The following preparations were performed on each sample before extraction. Samples stored at − 80 °C were thawed on ice and suspended in ASL buffer (Qiagen, Valencia, CA) at the following ratio: 2.5 mL ASL to 0.5 g of meconium, 1 mL of ASL to 0.2 g of stool, and 1 mL of ASL to one square of FOBT card [9–11]. For samples stored in ET tubes, the range of meconium used for each extraction was 0.1–0.5 g and the stool was 0.25–0.6 g. Tube-stored samples were aliquoted into 2 mL Matrix A lysis tubes (MP Biomedical, Santa Ana, CA), and FOBT samples were transferred to 2 mL Matrix B lysis tubes (MP Biomedical, Santa Ana, CA). The Matrix A tube was the preferred tube for meconium as it was found that with the Matrix B tube, beads were getting stuck in the sample and not homogenizing well; therefore, the Matrix A tube was used for the tube sample types because it worked more effectively with the meconium and kept the processes similar across tube samples. The Matrix A tube was not used for FOBT samples because it broke up the hemoccult paper excessively which was overcome by using the Matrix B tube. Samples were homogenized for 10 min on an oscillating vortexer at maximum speed and placed briefly in a flash spinner [9–13]. Twenty-five microliters of lysozyme at 20 mg/mL was added to tubes and inverted 10 times to mix [8]. Samples were placed on a shaking heat block at 95 °C for 5 min at 2000 rpm, cooled on ice for 2–5 min, and centrifuged at 20,000×*g* for 2 min. Supernatant was removed, avoiding the pellet, and placed in a new 2-mL ET [8, 10, 11] with one Inhibit X tablet (Qiagen, Valencia, CA). Samples were vortexed until the tablet was dissolved and incubated at room temperature for 3 min [11, 14]. Samples were centrifuged at 20,000×*g* for 2 min; supernatant was removed and put into a clean 1.5-mL ET and centrifuged again at 20,000×*g* for 3 min [11].

The Qiagen QIAmp DNA Stool Mini kit protocol was followed for manually extracted samples (Qiagen, Valencia, CA). For automated extraction, 200 μl of supernatant were loaded onto the EZ1 Advanced XL (Qiagen) using the EZ1 Tissue Kit with the Bacteria DNA extraction protocol card. Once extractions were complete, samples were filtered through Zymo-Spin™ IV-HRC Columns (Zymo Research, Irvine, CA) and stored at − 80C until sequencing [14].

Literature suggested that the EZ1 tissue kit in conjunction with the bacteria extraction card produced suitable DNA yields for sequencing [10, 11]. Ideally, an automated workflow is preferred for extraction because of the possibility of high sample volumes in future studies. Stool samples stored in ETs were extracted using the EZ1 and then manually if sample volumes permitted. Samples collected on FOBT cards were extracted solely on the EZ1. Although at least duplicates were attempted to be sequenced for each storage condition, due to either low stool mass or a sample failing amplification for library preparation, multiple replicates were not always possible and there was a difference in the number of samples sequenced at each condition (Tables 1 and 2). Due to the potential bias introduced by extraction methods, we have excluded the stool samples extracted manually. Meconium samples stored in ETs were extracted manually and then on the EZ1 if there was enough sample volume. In order to demonstrate that meconium samples extracted using the EZ1 were comparable to the manually extracted tubes once sequenced, duplicates of each storage condition were extracted and sequenced if possible. Because of the large amount of meconium samples stored in ETs, multiple extractions from the same storage conditions were possible.

**Table 1:** Demographics of subjects and number of samples for each diaper of stool used in the analysis for each storage condition

Diaper ID	Age of subject (days)	Sex	FOBT card 3–5 days RT	FOBT card 7 days, − 80 °C	FOBT card 7 days RT	ET 0–2 days RT	ET 7 days, − 80 °C
S1	7	M	2	2	2	2	4
S4	791	F	2	2	1	2	8
S5	25	F	2	2	1	4	2
S7^a^	32	M	2	2	1	4	4
S9^a^	33	M	2	2	2	4	4
S10	8	M	2	2	2	4	4
S11	8	M	2	2	2	4	4
S13	5	F	2	1	2	3	2
All			16	15	13	27	32

^a^S7 and S9 are two diapers from the same child collected at different times

**Table 2:** Demographics of subjects and number of samples for each diaper of meconium used in the analysis for each storage condition

Diaper ID	Age of subject (days)	Sex	FOBT card 3–5 days RT	FOBT card 7 days RT	FOBT card 7 days, − 80 °C	ET 0–2 days RT	ET 7 days, −80 °C
M1	0	F	1	2	2	6	4
M2	1	M	1	1	1	4	1
M3	1	M	1	1	1	1	3
All			3	4	4	11	8

### Sequencing

Sequencing libraries were prepared using a Nextera XT kit (Illumina, San Diego, CA) according to the 16S Metagenomic Illumina Library Preparation Protocol for sequencing the variable V3 and V4 regions of the 16S rRNA gene. If samples failed QC, library preparation was completed again with the “PCR 1” using Hemo KlenTaq® (New England Biolabs, Ipswich, MA) for the PCR reaction. The reason this was done is because Hemo KlenTaq® is known to work well with sample containing PCR inhibitors, especially bilirubin which is highly present in meconium samples. If a sample failed QC again after the second round of library preparation, it was not sequenced. The number of replicates that could be sequenced for each specific storage condition is shown in Tables 1 and 2. Each specimen/condition passing QC was then sequenced with replicates on Illumina MiSeq V3 kit (Illumina, San Diego, CA) with paired-end reads 301 bp (600 cycles).

Sequencing of negative controls of lysis buffer undergoing the above DNA extraction process for both stool (manual extraction and EZ1 Tissue Kit) and meconium was performed.

### Data analysis

Reads were organized into Operational Taxonomic Units (OTUs) by QIIME 1.9 using the open reference method. Meconium and stool samples were analyzed separately because of their distinct characteristics in species diversity and microbiota composition [8, 15]. The tools and libraries used in this analysis are listed in Additional file 1: Table S1.

For stool and meconium samples, OTUs that were not seen more than three times in at least 20% of the samples were removed. After this procedure, a single rarefaction was performed at 10,754 reads for the meconium samples, which removed one sample with only 4621 reads (Additional file 1: Figure S1A). For the stool samples, a single rarefaction was performed at 45,083 reads, keeping all of the samples (Additional file 1: Figure S1B).

Four beta diversity measures were investigated, namely, unweighted UniFrac distance, weighted UniFrac distance, Bray–Curtis, and Jensen–Shannon divergence using the phyloseq and vegan packages in R. Principal coordinate analysis using these four measures was carried out using the phyloseq package. The Adonis method with 99,999 permutations was used to determine whether there were significant differences between individual diaper samples, between storage containers (FOBT cards vs. ETs), and between each storage condition based on unweighted pairwise UniFrac distances.

Four different alpha diversity measures (observed species, Shannon, Simpson, Fisher) were compared between the two containers (FOBT cards vs. ETs) and the five different storage conditions, as well as comparing these measures in each condition with those stored at − 80 °C for 7 days in an ET as well as fresh stool in an ET tube for < 2 days, using the approximative Fisher–Pitman test, stratified by individual sample IDs, with 9999 Monte Carlo permutations. The tube stored at − 80 °C is considered the current standard used in most studies to date.

To examine relative abundance, OTUs without an assigned genus were filtered out. Furthermore, in order to investigate the relative abundance of the richer orders, only those orders with a relative abundance of at least 10% in any samples were investigated.

The reproducibility of the sequencing from stool samples was investigated between the different storage containers/conditions in terms of the four different alpha diversity measures, first component of the PCoA analysis using unweighted UniFrac distance, and relative abundances of the two most abundant phylum (Firmicutes and Proteobacteria) using the intraclass correlation coefficient (ICC). The same procedure was followed as described by Flores et al. [16]; the technical replicates were averaged for each storage method/condition, then ICCs were calculated for the above metrics across different storage conditions, within each of the storage conditions, and between samples stored on FOBT cards for 7 days at room temperature and those stored in ETs for 7 days at − 80 °C. This analysis was carried out using the ICC package in R.

## Results

Three diapers with meconium and eight diapers with more mature stool were collected and aliquoted into the different storage conditions described. Subject age for stool samples ranged from 5 to 791 days (median 16.5 days). Subject demographics can be seen in Tables 1 and 2.

### Meconium and stool samples

PCoA analysis was performed based on the UniFrac distances of the assigned OTUs in all samples (meconium and stool samples combined, Additional file 1: Figure S2A and B). The Adonis test indicated that there was a significant difference between the meconium and stool samples (*P* < 10^− 5^); therefore, the two types of samples were separated for further analyses.

### Rarefaction and alpha diversity

Rarefaction plots for meconium and stool samples are shown in Additional file 1: Figure S1A and 1B. Meconium samples showed a higher number of assigned OTUs than stool samples with the same number of sequences at 10,000 (603 OTUs vs. 452 OTUs, *P* = 1.51 × 10^−6^, *t* test). Based on the rarefaction plots, a single rarefaction was performed at 10,754 sequences for the meconium samples and 45,083 sequences for the stool samples. Even at this depth, the difference in number of OTUs was still significant (564 OTUs vs. 369 OTUs, *P* = 5.07 × 10^−8^).

**Fig. 1:**
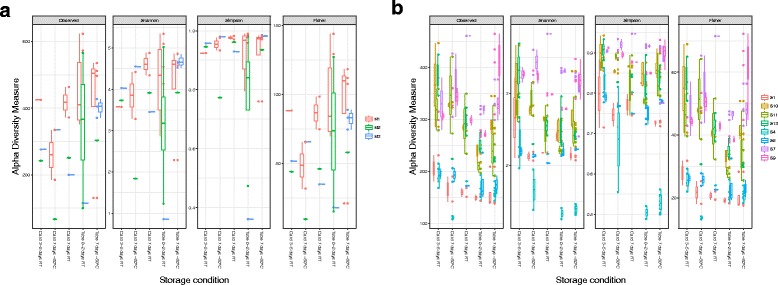
**a** Alpha diversities over storage methods in meconium samples. **b** Alpha diversities over storage methods in stool samples

Different alpha diversity measures across the different storage conditions are shown in Fig. 1a, b. The approximative Fisher–Pitman test statistic indicated that there were significant differences between different storage containers (all FOBT cards vs. all ETs) using all four alpha diversity measures in the stool samples but not in the meconium samples (Tables 3 and 4). While all four alpha diversity measures indicated richness of the samples, the Shannon index and Simpson index also measured the evenness of the sample. Further, the Simpson index gave more weight to the more abundant species. The samples stored in the tubes seemed to have lower Shannon and Simpson indexes; this could be due to that while the cards preserve the species present in the original sample, the relative abundance of these samples may have shifted slightly. Using the samples stored at − 80 °C in ETs for 7 days as the baseline for bacterial composition for the diapers being sampled, pairwise comparison was performed between the other four storage conditions with this baseline. Cards stored at room temperature for 7 days did not have significant differences in these alpha diversity measures when compared to ETs stored at − 80 °C for 7 days. All storage methods had significant differences in most alpha diversity measures when compared to ETs stored at room temperature for 0–2 days as a baseline.

**Table 3:** Comparison of alpha diversity measures among Meconium samples using the approximative Fisher–Pitman test stratified by individual sample IDs with 9999 permutations

	Observed	Shannon	Simpson	Fisher
Card vs. tube	0.06	1.00	0.27	0.05
Manual vs. EZ1	*0.04*	0.073	0.23	*0.033*
RT vs. − 80 °C	0.67	0.59	0.35	0.69
Tube (7 days at − 80 °C)^a^	0.76	0.22	0.13	0.81
Card (3–5 days)^a^	0.84	0.53	0.26	0.83
Card (7 days RT)^a^	0.69	0.25	0.18	0.61
Card (7 days at **−** 80 °C)^a^	0.14	0.93	0.44	0.14
Card (3–5 days)^b^	0.47	0.35	0.90	0.42
Card (7 days RT)^b^	0.44	0.91	0.89	0.41
Card (7 days at **−** 80 °C)^b^	0.13	0.93	0.44	0.14

^a^Comparison with tube at 0–2 days

^b^Comparison with tube 7 days at − 80 °C

Values italicized are signficiant with a value of p<0.05

**Table 4:** Comparison of alpha diversity measures among stool samples using the approximative Fisher–Pitman test stratified by individual sample IDs with 9999 permutations

	Observed	Shannon	Simpson	Fisher
Card vs. tube	*< 0.001*	*<0.001*	*< 0.001*	*0.001*
RT vs. − 80 °C	0.44	0.22	0.11	0.40
Tube (7 days at − 80 °C)^a^	*0.001*	0.47	0.96	*0.001*
Card (3–5 days)^a^	*0.01*	*0.02*	*0.04*	*0.01*
Card (7 days RT)^a^	*< 0.001*	*0.02*	0.06	*< 0.001*
Card (7 days at **−** 80 °C)^a^	*0.002*	*0.01*	*0.02*	*0.001*
Card (3–5 days)^b^	0.06	*< 0.001*	*< 0.001*	0.07
Card (7 days RT)^b^	0.52	0.24	0.05	0.55
Card (7 days at − 80 °C)^b^	0.56	0.07	*0.02*	0.54

^a^Comparison with tube at 0–2 days

^b^Comparison with tube 7 days at − 80 °C

Values italicized are signficiant with a value of p<0.05

### Beta diversity

**Fig. 2:**
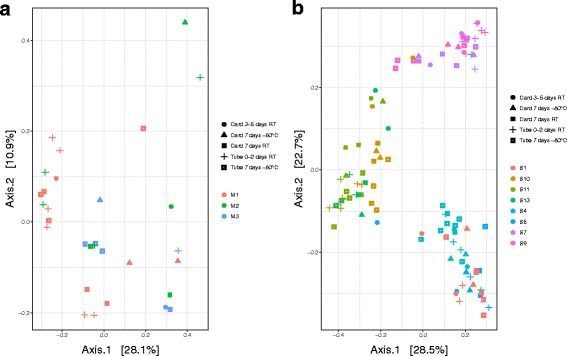
**a** PCoA plots based on pairwise UniFrac distances for meconium samples. **b** PCoA plot based on pairwise UniFrac distances for stool samples

**Fig. 3:**
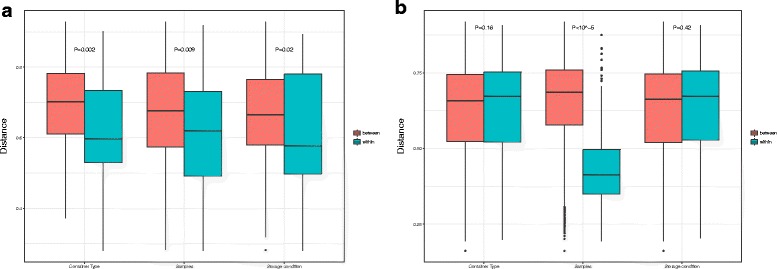
**a** Unweighted UniFrac distance between and within meconium samples. **b** Unweighted UniFrac distance between and within stool samples

Principal coordinate analysis (PCoA, Fig. 2a) based on unweighted UniFrac distances indicated that the meconium samples are mainly clustered by container (*P* = 0.002), followed by individual diaper (*P* = 0.009) and storage condition (*P* = 0.02) (Fig. 3a). Instead, the stool samples are clustered mainly by individual diaper (Fig. 2b 3 *P* < 10^− 5^, Adonis), rather than by storage condition (*P* = 0.42, Adonis) or container (*P* = 0.16, Adonis) (Fig. 3b) (Additional file 1: Table S3 and S4). PCoA analyses based on other distances (weighted UniFrac, Bray, and Jensen–Shannon divergence) show the same trend for both meconium and stool samples (Additional file 1: Figure S3).

Since two alpha diversities (observed OTU and Fisher diversity) are significantly different between manual and EZ1 extraction in meconium samples, we compared the unweighted UniFrac distances in extraction methods between fresh stool samples (tube with 0–2 days) and did not observed a significant difference (*P* = 0.11, Adonis).

Similar to the pairwise comparison with the baseline in alpha diversities, the unweighted UniFrac distances were compared between each of the storage condition with those stored in ETs at room temperature for 0–2 days in the stool samples. None of storage conditions showed significant difference compared with the baseline of ETs at room temperature for 0–2 days.

### Relative abundance

**Fig. 4:**
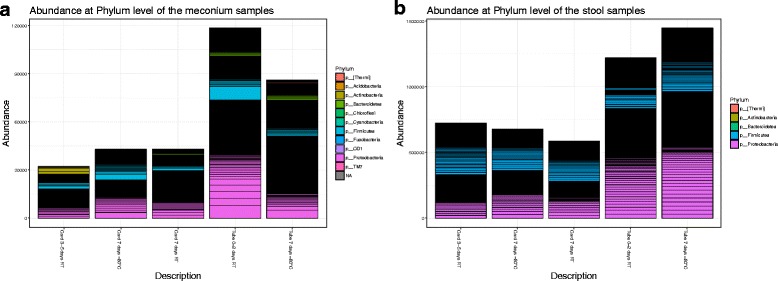
**a** Relative abundance at phylum level in meconium samples. **b** Relative abundance at phylum level in stool samples

Meconium and stool samples shared the same most abundant phyla (*Firmicutes* and *Proteobacteria*). These two phyla accounted for on average 86% for meconium samples and 99% in stool samples. There was no significant difference in relative abundance between storage containers (FOBT cards vs. ETs) in the meconium samples in these two phyla (*P* > 0.05, approximate Fisher–Pitman permutation test). On the other hand, the stool samples stored with cards tended to have an increase in *Firmicutes* and a reduction in *Proteobacteria* (*P* < 0.001 for *Firmicutes* and *P* < 0.001 for *Proteobacteria*) compared with ETs. A pairwise comparison was performed of the relative abundance of *Firmicutes* and *Proteobacteria* between samples stored at each condition with the baseline of those stored in ETs at − 80 °C for 7 days; none of the comparisons were significant after Bonferroni correction for meconium or stool (Fig. 4a, b).

The highest relative abundance for Order and Family were as follows: *Enterobacteriales* (33%, 46%) and *Enterobacteriaceae* (33%, 46%) for both meconium and stool respectively.

The relative abundance at the genus level was also investigated. Stool and meconium samples shared the top genus *Escherichia*, which accounted for 14% in stool and 6% in meconium. On the other hand, the second most abundant genus for stool was *Haemophilus* (11%) and *Pseudomonas* for meconium (5%). While there was no significant difference in the relative abundance of *Escherichia* between container types in the stool samples (*P* = 0.15), the relative abundance of *Haemophilus* was significantly different between the container types (*P* = 6 × 10^− 4^). Finally, there were no significant differences between container types in the two most abundant genera for meconium (complete OTU table in Additional file 1).

### Reproducibility and consistency

The estimation of reproducibility as measured by ICC in the stool samples is shown in Table 5. Different storage conditions and containers were reliable in preserving the microbial memberships (high ICCs in first component of PCoA analysis based on unweighted UniFrac distance) and slightly less reliable in preserving the relative microbial composition (moderate ICCs in relative abundance).

**Table 5:** Estimation of reproducibility as measured by intraclass correlation coefficient (ICC) in the stool samples

	All	Tube	Card	Card 7 days RT vs. tube 7 days, − 80 °C	Card 7 days RT vs. tube 0–2 days	Card 7 days, − 80 °C vs. tube 7 days, − 80 °C
Observed species	0.71	0.74	0.75	0.75	0.72	0.87
Shannon	0.74	0.96	0.71	0.75	0.78	0.92
Simpson	0.61	0.92	0.70	0.45	0.40	0.82
Fisher	0.70	0.72	0.74	0.73	0.69	0.86
PCoA1^a^	0.87	0.85	0.88	0.90	0.90	0.90
*Firmicutes*	0.51	0.38	0.87	0.60	0.09	0.72
*Proteobacteria*	0.53	0.39	0.88	0.62	0.13	0.73
*Esherichia*	0.80	1.00	0.84	0.72	0.71	0.92
*Haemophilus*	0.67	0.92	0.82	0.79	0.64	0.75

The “All” column measures the reproducibility of sample measures using all storage conditions. The “Tube” column measures the reproducibility of sample measures using the tube storage method with different lengths of time and temperatures. The “Card” column measures the reproducibility of sample measures using the card storage method with different lengths of time and temperatures. The last column shows the reproducibility measured by ICC between the same samples stored with cards at room temperature for 7 days compared with those stored with tubes at − 80C for 7 days

^a^First component from PCoA analysis based on unweighted UniFrac distance

### Negative controls

There was no detectable DNA using the Qubit® dsDNA HS Assay Kit (Life Technologies) machine before library preparation in any negative control sample. In the relative abundance analysis for stool, the statistical significance with just two samples could not be compared; however, it was noted that these two control samples seemed to have a much higher proportion of Actinobacteria at the phylum level. Specifically, the top 5 Phyla were as follows: Firmicutes (30%), Proteobacteria (26%), Actinobacteria (24%), Bacteroidetes (8.7%), and Thermi (5.2%). In contrast, Actinobacteria consisted of less than 0.5% in the test stool samples. The top 5 relative abundance at the genus level are as follows: Tepidimonas (13%), Corynebacterium (11%), Bifidobacterium (8%), Corynebacterium (7%), and Staphylococcus (7%). For the meconium control, the top 5 phyla were as follows: Actinobacteria (42%), Firmicutes (35%), Proteobacteria (18%), Bacteroidetes (5%), and Fusobacteria (0.1%). The relative abundance of Actinobacteria is less than 5% in the test meconium samples. The top 5 genera in the meconium control were as follows: Corynebacterium (21%), Kocuria (7%), Faecalibacterium (6%), Ruminococcus (5%), and Brevibacterium (3%).

## Discussion

It is crucial for large-scale prospective microbiota studies in infants and children to have a convenient and low-cost method for stool collection and storage such as FOBT cards that requires only a small amount of stool and that can still provide accurate sequencing data comparable to traditional methods. To our knowledge, this is the first study examining infant stool collected from diapers to show that microbiota structure based on unweighted UniFrac distances differed by individual stool sample but not by storage condition. There is no significant difference in alpha and beta diversity measures from 16S rRNA gene sequencing between stool stored on a FOBT card for 7 days compared to the current standard of stool frozen in an ET at − 80 °C. There were significant differences in microbiota structure however for meconium samples between the different storage conditions, with samples clustering mainly by container type rather than individual diaper.

A number of studies have looked at microbiota diversity and compositional stability across different storage methods in adults. Carroll et al. [17] showed that fecal samples stored at − 80 °C for 16S rRNA gene sequencing exhibit a microbial composition and diversity that shares more identity with its host of origin than any other sample and is currently the standard used in many studies. Both Dominianni et al. [1] and Sinha et al. [2] showed that microbiota profiles differed more between individuals than collection methods including FOBT cards and there was high reproducibility between collection methods, although there were some systematic biases according to sample methods [2]. However, other adult studies have shown that storage at ambient temperature [3, 18] can significantly affect the microbial composition of fecal samples. Pediatric stool samples, especially meconium and in early infancy, differ from adult stool samples both in microbiota diversity and composition [4, 8], and therefore, it is important to evaluate whether FOBT cards are still a reasonable method for collection and storage of infant stool.

Overall for infant stool samples, microbiota structure based on unweighted UniFrac distances differed most by individual stool sample rather than storage container or condition, as seen in adult studies [1, 2]. Similar to Dominianni et al. [1], differences were seen overall in stool samples stored on cards tending to have an increase in *Firmicutes* and a reduction in *Proteobacteria* compared with ET tubes. Conversely, in infant fecal samples, Shaw et al. [19] noted that at the level of phyla, longer storage at room temperature was associated with a significant decrease in *Firmicutes* and an increase in *Proteobacteria* in complex fecal communities. Taken together, these results do suggest different storage methods and containers may impact relative abundance of certain taxa and should be taken into account when interpreting results of a study. It is also possible that the use of different lysis tubes for ET and FOBT samples in this study may have contributed to the differences in taxa seen.

Concerns have been raised regarding the stability of DNA when samples are kept at room temperature [3, 18] and resulting effects on microbiotametrics. Interestingly, in infant fecal samples, Shaw et al. [19] found that storing samples at room temperature does lead to significant changes in the microbial community, both in alpha and beta diversity measures and for specific phyla after 2 days. This differs from the results of our study where FOBT cards for 7 days at RT were comparable in beta diversity measures and also in relative abundance of major phyla with fresh stool samples stored in an ET tube. The reason for these differing results is unclear, although storage containers/conditions varied between the studies, and certainly warrants further investigation. In addition, Guo Y et al. [20] found that exposure to room temperature even for a short period of time (up to 2 h) before freezing resulted in changes to some specific taxa, although overall microbiota composition appeared stable; our study was not designed to detect possible changes to the microbiota with short-term exposure to room temperature.

Interestingly for meconium samples, microbiota structure based on unweighted UniFrac distances did differ, with clustering of samples based on storage container and condition, not just by individual diaper. Therefore, we would be hesitant to use FOBT cards for the collection and storage of meconium samples for microbiota analysis. The reason for this difference of meconium compared with infant stool is not entirely clear, however may be due to the overall microbiota composition varying from stool [8], with lower bacterial concentrations. Additionally, the physical consistency of meconium varies from stool and is often much “stickier”; the stickiness of the sample makes it harder to break apart the matrix and get access to the bacterial cells and therefore developing an effective DNA extraction method for meconium as done in this study may create bias. However, other measures in alpha diversity and relative abundance of major phyla were similar in meconium samples across different storage conditions and containers.

Importantly for infants, often, only very small stool samples can be collected and scraped from the diaper. Here, we showed that even with stool samples as small as 0.013 g on a FOBT card, adequate quality DNA could be extracted and sequenced. Supporting this, Shaw et al. [19] when looking at stool samples as small as 0.025 g found no significant associations between reduced sample size and shifts in any measure of alpha diversity and most beta diversity measures. Despite this, it would likely be wise to establish a minimum sample size for stool for DNA extraction on a study by study basis to give consistent results.

Overall, our finding show that the different storage conditions/containers are reliable in preserving the microbial memberships (high ICCs in first component of PCoA analysis based on unweighted UniFrac distance) and slightly less reliable in preserving the relative microbial composition (moderate ICCs in relative abundance). If these differences and limitations are acknowledged in study design and interpretation, FOBT cards may represent a convenient and effective method for longitudinal large-scale outpatient stool microbiota studies in children where samples need to be mailed back. Given the differences seen, however, the same sample collection and storage method should be used within a single study.

One limitation of this study is the microbiota was only analyzed by 16S rRNA gene sequencing; shotgun metagenomic sequencing is becoming increasingly used in studies for a comprehensive view of the fecal microbiota, and it is warranted to see if storage on FOBT cards still produce reasonable results with differing methods of sequencing. Additionally, stool was applied carefully to FOBT cards by a single experienced laboratory technician. In the “real world,” there may be discrepancy between parents of subjects in a study about how a stool sample from a diaper is applied to FOBT card before mailing (although clear instructions can be given) and our study does not account for the potential differences between the person applying the stool. Our study includes infants of different ages and in different stages of microbiota development; this was desired to investigate a range of ages; however, for a validation study, looking at infants all at the same age may have resulted in less variation due to the dynamic microbiota changes that occur in early life. There are also a differing number of replicates of each sample based on the amount of stool available; however, this was accounted for in the analysis. Additionally, this study is looking at infants who are in a stable state of health; microbiota studies in children often include ill children with differing states of dysbiosis and it is unknown if FOBT cards would be as suitable in these conditions. Lastly, although samples stored at − 80 °C on a FOBT card were examined, these were only held at this temperature for a week. In many studies, stool samples remain frozen for several months or years and it would not be practical to sequence samples within a 7-day period. It has been shown that microbiota analysis from infant fecal samples frozen for 2 years in an ET leads to few significant changes in the microbial community [19]; however, it is unknown how stable a FOBT card at − 80 °C will remain overtime. The results from this study cannot currently validate using FOBT cards that have been stored for longer time periods, and further studies are needed to compare the reproducibility of sequencing results from FOBT cards stored at − 80 °C compared with stool frozen in an ET after long-term storage.

## Conclusion

The feasibility of using FOBT cards as a convenient alternative to collect and store infant stool and meconium samples compared to freezing stool in ETs for 16S rRNA gene sequencing was investigated. Infant stools did cluster mainly by individual stool sample based on unweighted UniFrac distances, rather than storage condition or container, and there was no difference in alpha and beta diversity measures between stool stored on a FOBT card at room temperature for 7 days and a “gold standard” of fresh stool stored in an ET. Overall, all the different storage methods/conditions were reliable in preserving the microbial memberships and slightly less reliable in preserving the alpha diversity and relative microbial composition of infant stool, and these differences must be taken into account when designing and interpreting a study. Moreover, ideally, the same storage condition should be used across an entire study for consistency. Conversely, meconium samples did cluster by container more than individual diaper sample based on unweighted UniFrac distances suggesting accuracy of sequencing is not preserved when stored on FOBT cards. Hence, acknowledging certain limitations, FOBT cards may be a useful tool in large-scale stool microbiota studies in children requiring outpatient follow-up but should not be used when studying meconium.

## Additional file

Additional file 1: Table S1: Software and R libraries used in the analysis. Table S2: *P* values from Adonis tests for different contrasts with different rarefaction cutoffs and different distance measures. Figure S1A: Rarefaction plot of number of assigned OTUs versus number of sequences from different storage conditions in meconium samples. The vertical line indicates the number of sequences used for the single rarefaction in analyses. Figure S1B: Rarefaction plot of number of assigned OTUs versus number of sequences from different storage conditions in stool samples. The vertical line indicates the number of sequences used for the single rarefaction in analyses. Figure S2: PCoA plots for all samples. Figure S3A. PCoA plots with Bray–Curtis, Jensen–Shannon divergence, unweighted UniFrac, and weighted UniFrac distance measures for meconium samples. Figure S3B. PCoA plots with Bray–Curtis, Jensen–Shannon divergence, unweighted UniFrac, and weighted UniFrac distance measures for stool samples. Compressed OTU file with all the samples at Family level: stoolAndMeconiumOTUByFamily.csv.zip. Compressed OTU file with all the samples at Genus level: stoolAndMeconiumOTUByGenus.csv.zip. (ZIP 3263 kb)
