# Supplementary material for: Collection of non-meconium stool on fecal occult blood cards is an effective method for fecal microbiota studies in infants
Source: Microbiome. 2017 Sep 5;5:114. doi: 10.1186/s40168-017-0333-z (PMC5583988; doi:10.1186/s40168-017-0333-z)
Supplement: Additional file 1: — Table S1: Software and R libraries used in the analysis. Table S2: P values from Adonis tests for different contrasts with different rarefaction cutoffs and different distance measures. Figure S1A: Rarefaction plot of number of assigned OTUs versus number of sequences from different storage conditions in meconium samples. The vertical line indicates the number of sequences used for the single rarefaction in analyses. Figure S1B: Rarefaction plot of number of assigned OTUs versus number of sequences from different storage conditions in stool samples. The vertical line indicates the number of sequences used for the single rarefaction in analyses. Figure S2: PCoA plots for all samples. Figure S3A. PCoA plots with Bray–Curtis, Jensen–Shannon divergence, unweighted UniFrac, and weighted UniFrac distance measures for meconium samples. Figure S3B. PCoA plots with Bray–Curtis, Jensen–Shannon divergence, unweighted UniFrac, and weighted UniFrac distance measures for stool samples. Compressed OTU file with all the samples at Family level: stoolAndMeconiumOTUByFamily.csv.zip. Compressed OTU file with all the samples at Genus level: stoolAndMeconiumOTUByGenus.csv.zip. (ZIP 3263 kb) [file 40168_2017_333_MOESM1_ESM.zip › SupplementaryInformation 8-1.docx]

# Supplementary Information

## Supplementary Table 1: Software and R libraries used in the analysis

## Supplementary Table 2: P values from Adonis tests for different contrasts with different rarefaction cutoffs and different distance measures

Supplementary Figure 1A: Rarefaction plot of number of assigned OTUs versus number of sequences from different storage conditions in meconium samples. The vertical line indicates the number of sequences used for the single rarefaction in analyses.

Supplementary Figure 1B: Rarefaction plot of number of assigned OTUs versus number of sequences from different storage conditions in stool samples. The vertical line indicates the number of sequences used for the single rarefaction in analyses.

Supplementary Figure 2: PCoA plots for all samples.

Supplementary Figure 3A. PCoA plots with bray-curtis, Jensen–Shannon divergence, unweighted unifrac and weighted unifrac distance measures for meconium samples.

Supplementary Figure 3B. PCoA plots with bray-curtis, Jensen–Shannon divergence, unweighted unifrac and weighted unifrac distance measures for stool samples.

Supplementary File 1. Compressed OTU file with all the samples at Family level: stoolAndMeconiumOTUByFamily.csv.zip.

Supplementary File 2. Compressed OTU file with all the samples at Genus level: stoolAndMeconiumOTUByGenus.csv.zip.

## Supplementary Table 1: Software and R libraries used in the analysis

| Tool | Version |
| --- | --- |
| Qiime | 1.9 |
| R | 3.3.1 |
| phyloseq | 1.18.0 |
| ggplot2 | 2.1.0 |
| cluster | 2.0.5 |
| igraph | 1.0.1 |
| markovchain | 0.6.5.1 |
| RColorBrewer | 1.1.2 |
| gridExtra | 2.2.1 |
| plyr | 1.8.4 |
| coin | 1.1.2 |
| ICC | 2.3.0 |
| vegan | 2.4.1 |
| reshape2 | 1.4.2 |
| cowplot | 0.6.3 |

## Supplementary Table 2: P values from Adonis tests for different contrasts with different rarefaction cutoffs and different distance measures in stool samples

| **comparison\rarefacation** | **Distance measure** | **3000** | **6000** | **10000** |
| --- | --- | --- | --- | --- |
| **Card vs. tube** | Unweighted unifrac | 0.16 | 0.24 | 0.25 |
|  | Bray | 0.15 | 0.17 | 0.17 |
| **Storage condition** | Unweighted unifrac | 0.42 | 0.51 | 0.58 |
|  | Bray | 0.52 | 0.54 | 0.55 |
| **Sample** | Unweighted unifrac | 10^-5^ | 10^-5^ | 10^-5^ |
|  | Bray | 10^-5^ | 10^-5^ | 10^-5^ |

Supplementary Figure 1: Rarefaction plots of number of assigned OTUs versus number of sequences

Supplementary Figure 1A: Rarefaction plot of number of assigned OTUs versus number of sequences from different storage conditions in meconium samples. The vertical line indicates the number of sequences used for the single rarefaction in analyses.

Supplementary Figure 1B: Rarefaction plot of number of assigned OTUs versus number of sequences from different storage conditions in stool samples. The vertical line indicates the number of sequences used for the single rarefaction in analyses.

## Supplementary Figure 2 PCoA plots for all samples


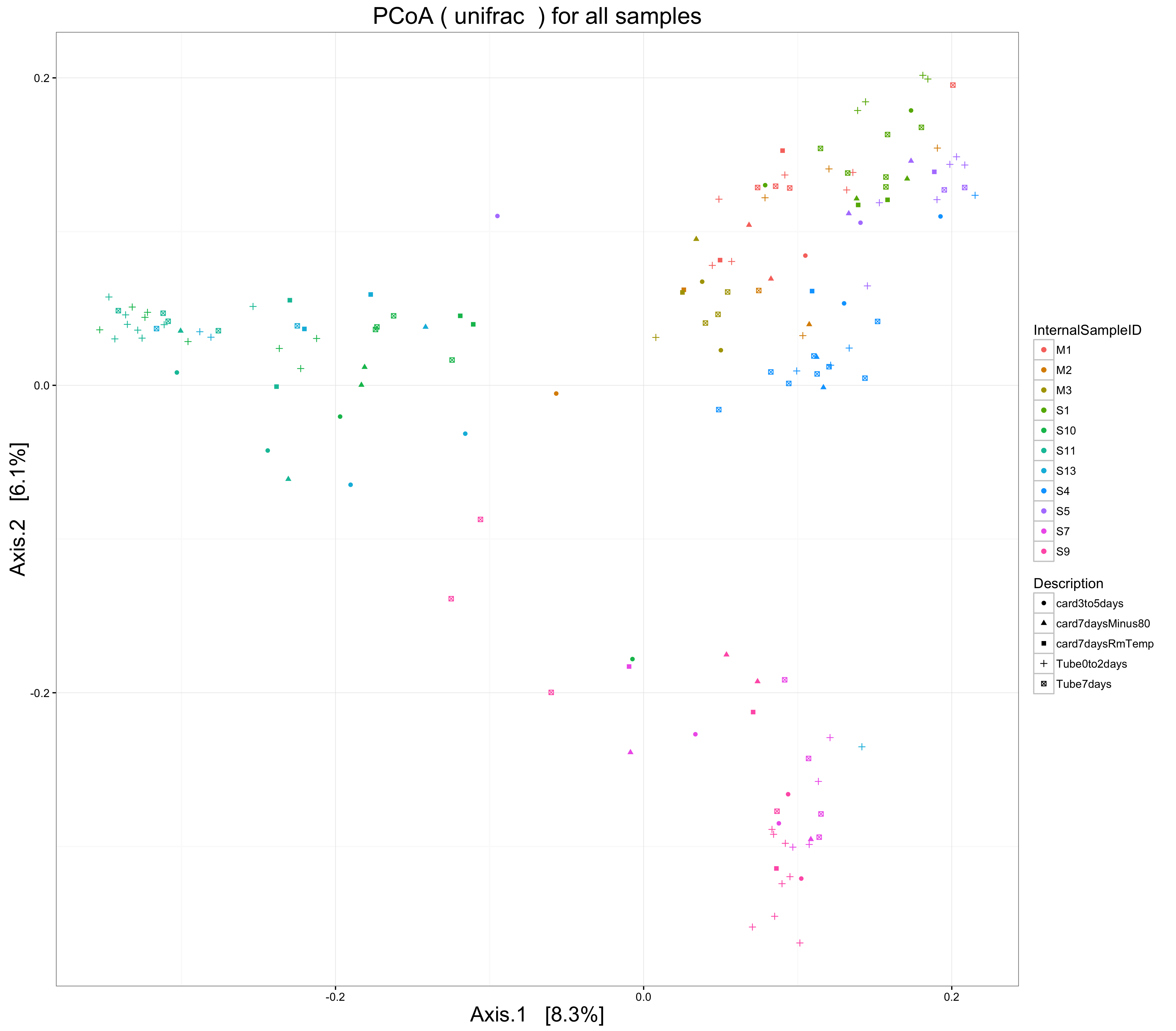


## Supplementary Figure 3 PCoA plots with bray-curtis, Jensen–Shannon divergence, unweighted unifrac and weighted unifrac distance measures.


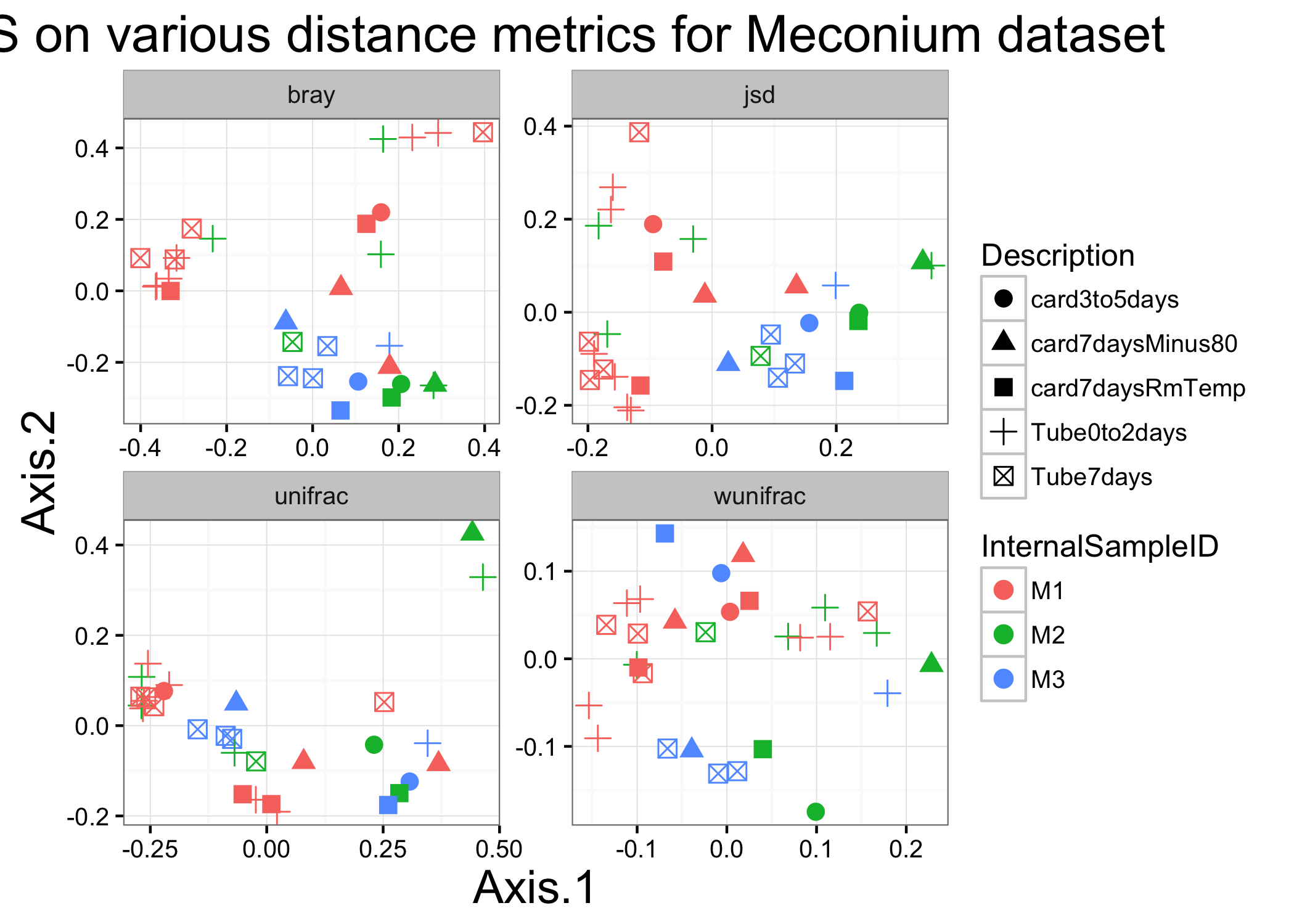


Supplementary Figure 3A. PCoA plots with bray-curtis, Jensen–Shannon divergence, unweighted unifrac and weighted unifrac distance measures for meconium samples.


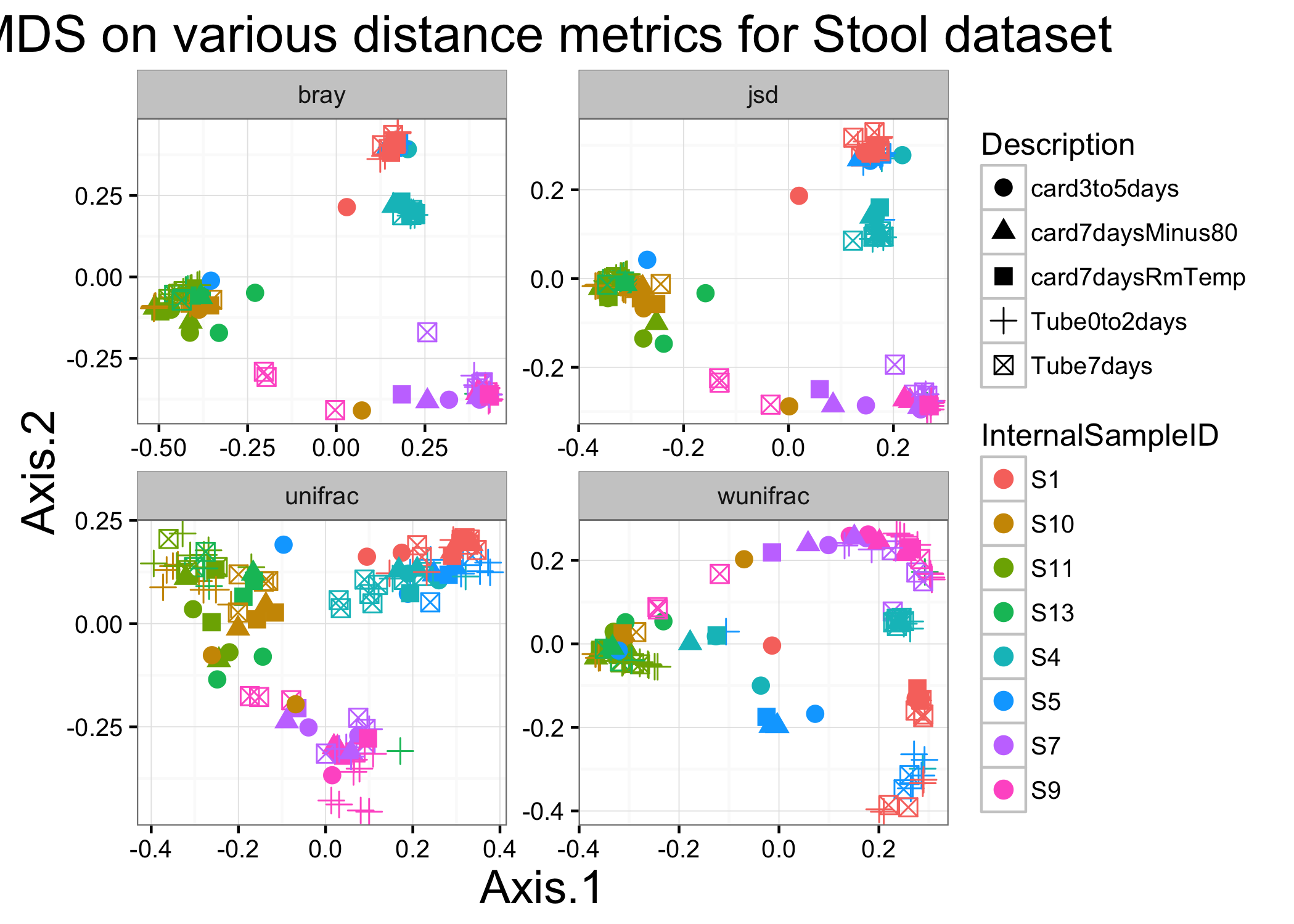


Supplementary Figure 3B. PCoA plots with bray-curtis, Jensen–Shannon divergence, unweighted unifrac and weighted unifrac distance measures for stool samples.
